# Meiotic Heterogeneity of Trivalent Structure and Interchromosomal Effect in Blastocysts With Robertsonian Translocations

**DOI:** 10.3389/fgene.2021.609563

**Published:** 2021-02-16

**Authors:** Shuo Zhang, Caixia Lei, Junping Wu, Jing Zhou, Min Xiao, Saijuan Zhu, Yanping Xi, Jing Fu, Yijuan Sun, Congjian Xu, Xiaoxi Sun

**Affiliations:** ^1^Shanghai Ji Ai Genetics and IVF Institute, Obstetrics and Gynecology Hospital, Fudan University, Shanghai, China; ^2^Key Laboratory of Female Reproductive Endocrine Related Diseases, Obstetrics and Gynecology Hospital, Fudan University, Shanghai, China

**Keywords:** Robertsonian translocation, trivalent structure, meiotic heterogeneity, segregation patterns, ICE

## Abstract

**Background:**

Robertsonian translocations are common structural rearrangements and confer an increased genetic reproductive risk due to the formation of trivalent structure during meiosis. Studies on trivalent structure show meiotic heterogeneity between different translocation carriers, although the factors causing heterogeneity have not been well elaborated in blastocysts. It is also not yet known whether interchromosomal effect (ICE) phenomenon occurs in comparison with suitable non-translocation control patients. Herein, we aimed to evaluate the factors that cause meiotic heterogeneity of trivalent structure and the ICE phenomenon.

**Methods:**

We designed a retrospective study, comprising 217 Robertsonian translocation carriers and 134 patients with the risk of transmitting monogenic inherited disorders (RTMIDs) that underwent preimplantation genetic testing (PGT). Data was collected between March 2014 and December 2019. The segregation products of trivalent structure were analyzed based on the carrier’s gender, age and translocation type. In addition, to analyze ICE phenomenon, aneuploidy abnormalities of non-translocation chromosomes from Robertsonian translocation carriers were compared with those from patients with RTMIDs.

**Results:**

We found that the percentage of male carriers with alternate segregation pattern was significantly higher [*P* < 0.001, odds ratio (OR) = 2.95] than that in female carriers, while the percentage of adjacent segregation pattern was lower (*P* < 0.001, OR = 0.33). By contrast, no difference was observed between young and older carriers when performing stratified analysis by age. Furthermore, segregation pattern was associated with the D;G chromosomes involved in Robertsonian translocation: the rate of alternate segregation pattern in Rob(13;14) carriers was significantly higher (*P* = 0.010, OR = 1.74) than that in Rob(14;21) carriers, whereas the rate of adjacent segregation pattern was lower (*P* = 0.032, OR = 0.63). Moreover, the results revealed that the trivalent structure could significantly increase the frequencies of chromosome aneuploidies 1.30 times in Robertsonian translocation carriers compared with patients with RTMIDs (*P* = 0.026), especially for the male and young subgroups (*P* = 0.030, OR = 1.35 and *P* = 0.012, OR = 1.40), while the mosaic aneuploidy abnormalities presented no statistical difference.

**Conclusions:**

Our study demonstrated that meiotic segregation heterogeneity of trivalent structure is associated with the carrier’s gender and translocation type, and it is independent of carrier’s age. ICE phenomenon exists during meiosis and then increases the frequencies of additional chromosome abnormalities.

## Introduction

Robertsonian translocations are one of the most common structural chromosomal rearrangements, consisting of a fusion of the long arms of two acrocentric chromosomes. In humans, this rearrangement occurs in the five pairs of acrocentric chromosomes, comprising chromosomes 13, 14, and 15 (group D) and 21 and 22 (group G). The prevalence of Robertsonian translocations is approximately 1.23/1000 in newborns (Nielsen and Wohlert, 1991), and 0.65 to 2.17% in couples with recurrent miscarriage (Stern et al., 1999; Dong et al., 2019). Although translocation can involve any two of these chromosomes, the most frequent ones are between chromosomes 13 and 14 and between chromosomes 14 and 21 (Anton et al., 2010). Although carriers of Robertsonian translocations are phenotypically normal, they produce abnormal gametes due to meiotic disjunction. As a consequence, many suffer from fertility problems and have an increased risk of chromosomally abnormal pregnancies that result in spontaneous abortion or the birth of babies with congenital anomalies (Godo et al., 2015; Mayeur et al., 2019).

During prophase I, the derivative chromosome and the two respective normal ones form a trivalent structure by homologous synapsis. At anaphase I, this structure could segregate according to three theoretical modes: (1) Alternate: the two non-translocated chromosomes segregate to the same pole, while the derivative moves to the other; (2) Adjacent: the derivative and one of the non-translocated chromosomes segregate to the same pole, while the remaining non-translocated one goes to the other; and (3) 3:0: the derivative and two non-translocated chromosomes move to the same pole (Scriven et al., 1998). Theoretically, gametes with eight different karyotypes are produced, although only two result from alternate segregation have normal or balanced karyotypes. The others from adjacent and 3:0 segregations have unbalanced nullisomic or disomic karyotypes (Scriven et al., 1998). With the development of assisted reproductive technology, preimplantation genetic testing for structural rearrangements (PGT-SR) was applied to Robertsonian translocation carriers and promising reproductive outcomes achieved by selective transfer of euploid embryos (Deleye et al., 2015; Ghevaria et al., 2016; Wang et al., 2018; Fodina et al., 2019).

Studies of the meiosis segregation patterns of the trivalent structure were performed on sperm with fluorescence *in situ* hybridization (FISH). Previous studies indicate that alternate segregation products are the most common, ranging from 68.0 to 94.4% of all segregation products, followed by adjacent, and 3:0 segregation products (Mahjoub et al., 2011; Bernicot et al., 2012; Pylyp et al., 2013; Kirkpatrick et al., 2015; Wang et al., 2017). Some studies reported that the translocationoutcomes showed heterogeneity between different translocation carriers’ gender. By analyzing the meiosis segregation products of the trivalent structure in cleavage-stage embryos with FISH, male carriers tended to produce more embryos with euploid products than female carriers (Bint et al., 2011; Ko et al., 2013). Furthermore, Zhang et al. (2019a) analyzed blastocysts with array comparative genomic hybridization (array-CGH), and found that the proportions of the trivalent’s meiotic segregation pattern differed significantly according to the carrier’s gender and were independent of the carrier’s age. On the contrary, Huang et al. (2010) concluded that genders of the carriers and different translocation types had no effect on the meiotic division patterns of the trivalent structure.

Previous studies have reported that Robertsonian translocation carriers can produce increased number of gametes with aneuploidy abnormalities, which are not related to the rearrangement and the phenomenon is defined as interchromosomal effect (ICE) (Guichaoua et al., 1990; Alfarawati et al., 2012). The ICE phenomenon is considered a consequence of disturbances in the correct disjunction of structurally normal chromosome pairs by the formation of specific pairing structures (Alfarawati et al., 2012). Hajlaoui et al. (2018) reported that ICE was observed by sperm FISH studies and contributed to higher rates of abnormal prenatal aneuploidy in sperm, then increasing the probability of chromosomal numerical abnormalities in embryos. Wiland et al. (2020) found most infertile Robertsonian carriers exhibited an increased frequency of aneuploidy that was not involved in a particular translocation in sperm and might result in the abnormal development of embryos. In addition, Gianaroli et al. (2002) obtained similar results by analyzing day 3 embryos of Robertsonian translocation carriers with FISH technology. On the contrary, Munne et al. (2005) suggested that ICE was negligible in embryos of Robertsonian translocation carriers and the other two researches suggested that ICE was absent (Tulay et al., 2015; Xie et al., 2018). In this field, there are few related articles and the results are inconsistent.

The present study aims to investigate potential factors that cause the meiotic segregation heterogeneity of trivalent structure and whether ICE occurs during meiosis with comprehensive chromosome screening (CCS) method. The analysis of the potential impact factors on meiotic segregation was conducted according to the carrier’s gender, carrier’s age and translocation type. Additionally, ICE was analyzed by comparing aneuploidy abnormalities of non-translocation chromosomes in blastocysts from Robertsonian carriers with those of patients with RTMIDs, including aneuploidy and mosaic aneuploidy abnormalities.

## Materials and Methods

### Study Subjects

In this study, a total of 217 Robertsonian translocation carriers and 134 patients with RTMIDs undergoing PGT were collected in Obstetrics and Gynecology Hospital of Fudan University affiliated Shanghai JI AI Genetics and IVF center from March 2014 and December 2019. These translocation carriers had a history of spontaneous abortion, infertility or pregnancy with chromosome anomalies. Karyotype analyses of the cultured blood lymphocytes were performed with conventional G-banding. The carriers of complex chromosome rearrangements were excluded. All the spouses of the translocation carriers, and all the patients with RTMIDs all had normal karyotypes. Written informed consent was obtained from each family before the start of PGT cycle. The study protocol was approved by the Ethics Committee for Human Subject research of Ji Ai Genetics & IVF Institute (JIAI-2018-16).

### IVF, Blastocyst Biopsy and Whole Genomic Amplification

Standard techniques were applied in *in vitro* fertilization (IVF) in Shanghai Ji Ai Genetics & IVF Institute of Obstetrics and Gynecology Hospital Fudan University. Briefly, retrieved MII oocytes were produced through intracytoplasmic sperm injection (ICSI), and then were cultured for 5–6 days so as to develop to the blastocyst stage. The grading criteria of blastocysts were in accordance with the recommendation by Schoolcraft et al. (1999). As per these criteria, three to ten trophoblast cells were biopsied and immediately transported to polymerase chain reaction tubes with an alkaline denaturation buffer for cell lysis. Whole genomic amplification (WGA) was performed by employing the multiple displacement amplification (MDA) method. Isothermal DNA amplification with phi 29 DNA polymerase (Repli-g single cell kit, QIAGEN GmbH, Hilden, Germany) was performed as the manufacturers’ protocol. The isothermal amplification incubated at 30°C for 8 h and the reaction was stopped by 65°C for 3 min.

### SNP-Array and Analysis

SNP genotypes of WGA products were conducted with Illumina Human Cyto-12v2.1 microarray for translocation carriers and Karyomap-12 microarray for monogenic disorder patients. Each bead chip contained approximately 300,000 SNPs. The experiment was carried out using the infinium procedure as previously described (Fan et al., 2005). Briefly, the WGA product was amplified again in an overnight isothermal reaction and then fragmented to a size of 300∼500 base pairs. After isopropanol precipitation and resuspension, the samples were hybridized to a bead chip overnight for about 20 h. Subsequently, an automated Extension Staining process was conducted. After staining, the bead chips were scanned on an iScan reader. Genotypes and molecular karyotypes were analyzed with GenomeStudio and KaryoStudio software (Illumina, Inc., San Diego, United States). Molecular karyotypes were used to identify chromosomal copy number variations (CNVs), including aneuploidy, segmental aneuploidy and mosaicism abnormalities.

### Statistical Analysis

Quantitative data with a normal distribution were reported with the mean ± standard deviation and were compared with a two sample Student’s *t* test. Data with a skewed distribution were expressed with the median and quartile range, which were compared with a Mann–Whitney *U* test. Categorical data were expressed as a frequency. The differences between the frequency distributions of segregation products were compared with χ^2^ or Fisher’s Exact Test. Stratified analysis was conducted to evaluate potential interaction by gender or age. All P values presented were two-sided, and the level of *P* < 0.05 was considered significant. All statistical procedures were conducted with SPSS version 17.0 software (SPSS, Chicago, IL, United States).

## Results

### Clinical Characteristics and Results

A total of 217 Robertsonian translocation carriers and 134 patients with RTMIDs were involved in the current study. Trophectoderm cell biopsy was carried out on 1,787 day 5/6 embryos. Among all the biopsied embryos, 1762 were successfully diagnosed with SNP-array method (98.60%), 504 (51.6%) were identified as normal or balanced from translocation carriers and 310 (39.5%) were identified as transferable from monogenic disorder patients. The diploid rate (57.4%) in male carriers was significantly higher than that (45.2%) in female carriers (*P* = 0.037, OR = 1.27). There existed no statistical difference in other aspects of clinical parameter between male and female translocation carriers (Table 1).

**TABLE 1:** Clinical characteristics and results of patients undergoing PGT with SNP array.

Parameter	Male translocation carriers	Female translocation carries	Monogenic disorder patients
Patients	99	118	134
Cycles	117	142	177
Female age (years)	30.3 ± 4.3	30.9 ± 4.0	33.2 ± 4.3
Male age (years)	32.3 ± 5.4	33.0 ± 5.7	35.2 ± 4.5
Retrieved oocytes	12.9 ± 6.8	11.5 ± 6.7	13.5 ± 7.6
Injected oocytes	11.1 ± 5.9	9.9 ± 5.7	11.4 ± 7.0
2-pronuclei zygotes	9.6 ± 5.4	8.8 ± 5.0	9.7 ± 6.2
D3 embryos	8.1 ± 4.8	7.0 ± 4.2	7.5 ± 5.0
Biopsied embryos	4.0 ± 3.0	3.4 ± 2.0	4.3 ± 3.6
Diagnosed embryos	4.0 ± 3.0	3.4 ± 2.0	4.0 ± 3.2
Transferable/normal embryos	2.3 ± 2.1	1.5 ± 1.1	1.8 ± 2.2
Abnormal embryos	1.7 ± 1.3	1.8 ± 1.6	2.2 ± 1.9
ET cycles	69	86	75
Positive HCG (%)	38(55.1%)	47(54.7)	44(58.7%)
Clinical pregnancies (%)	33(47.8%)	41(47.7%)	38(50.6%)
Spontaneous abortions (%)	4(5.8%)	4(4.7%)	4(5.3%)
Deliveries (%)	20(29.0%)	27(31.4%)	18(24.0%)
Ongoing pregnancies (%)	9(13.0%)	10(11.6%)	16(21.3%)

### Sperm Parameters

The parameters of sperm concentration and sperm progressive motility were lower in male carriers than those in the spouse of female translocation carriers (*p* = 0.022 and *p* = 0.015, respectively). However, the statistical difference was not observed in semen volume, total sperm and total progressively motile sperm (Supplementary Table 1).

### Embryo Meiotic Segregation Patterns of Trivalent Structure

**FIGURE 1:**
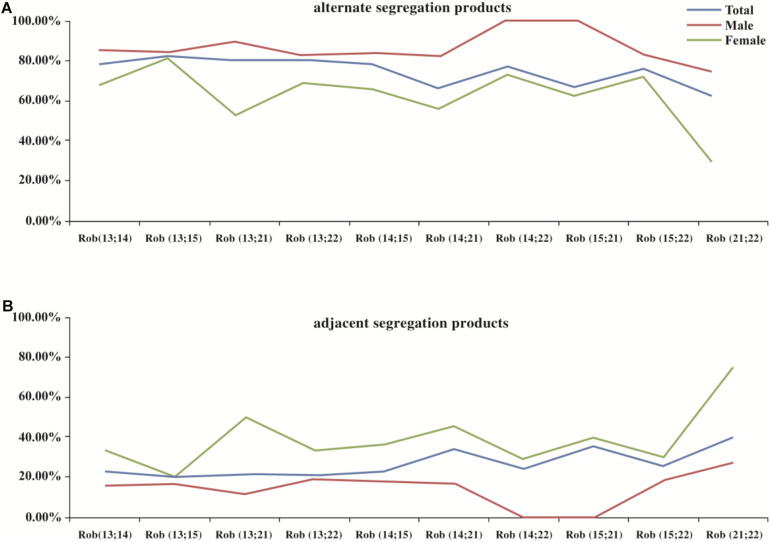
The distribution of segregation products in blastocysts among different translocation karyotypes. Legend: **(A)** represents the distribution of alternate segregation products; **(B)** represents the distribution of adjacent segregation products. Horizontal axis represents different translocation types, Rob(13;14)(q10;q10); Rob(13;15)(q10;q10); Rob(13;21)(q10;q10); Rob (13;22)(q10;q10); Rob(14;15)(q10;q10); Rob(14;21)(q10;q10); Rob(14;22)(q10;q10); Rob(15;21)(q10;q10); Rob(15;22)(q10;q10); and Rob (21;22)(q10;q10), respectively.

The meiotic segregation pattern was determined by molecular karyotype of embryos. In total 708 embryos were produced by alternate segregation pattern, which was the most common pattern (708/977, 72.5%), followed by adjacent (263/977, 26.9%) and 3:0/other segregation pattern (6/977, 0.6%). Figure 1 illustrated the distribution of segregation products from different translocation karyotypes.

When comparing the meiotic segregation products of trivalent structure in blastocysts between males and females, we found that the percentage of alternate segregation pattern in male carriers was significantly higher (*P* < 0.001, OR = 2.95) than that in female carriers, whereas the frequency of adjacent segregation pattern was lower (*P* < 0.001, OR = 0.33). In contrast, no statistical difference was observed between 3:0/other products (Table 2). Through carrying out further stratified analysis by age, the distribution of segregation products was roughly similar to the whole. When conducting the comparison of segregation products in embryos by carrier’s age, no difference was found between all these three segregation models (Table 3). Through conducting further stratified analysis by gender, we found no significant difference in the distribution. In order to further identify the effect of gender and age on different translocation types, we found that the results of segregation product in Rob(13;14) carriers, the most common translocation type, were almost equivalent to the overall cohort (Supplementary Tables 2, 3).

**TABLE 2:** Analysis of meiotic segregation patterns of trivalent structure in blastocysts according to carrier’s gender.

Segregation	Total	Carrier’s gender			Carrier’s gender (Zhang et al., 2019a)			*P* value^*a*^
Patterns		Male *n* (%)	Female *n* (%)	*P* value	Male *n* (%)	Female *n* (%)	*P* value	
Overall	977	473	504	***P* < 0.001**	280	324	***P* < 0.001**	NS
Alternate	708	390(82.45%)	318(63.10%)	***P* < 0.001**	232(82.86%)	179(55.25%)	***P* < 0.001**	
Adjacent	263	81(17.12%)	182(36.11%)	***P* < 0.001**	47(16.79%)	138(42.59%)	***P* < 0.001**	
3:0/others	6	2(0.42%)	4(0.79%)	NS	1(0.36%)	7(2.16%)	NS	
**Age < 35 years**								
Overall	836	421	415	***P* < 0.001**	195	288	***P* < 0.001**	NS
Alternate	610	348(82.66%)	262(63.13%)	***P* < 0.001**	162(83.08%)	160(55.55%)	***P* < 0.001**	
Adjacent	221	71(16.86%)	150(36.14%)	***P* < 0.001**	33(16.92%)	122(42.36%)	***P* < 0.001**	
3:0/others	5	2(0.48%)	3(0.72%)	NS	0(0.0%)	6(2.08%)	NS	
**Age ≥ 35 years**								
Overall	141	52	89	***P* = 0.023**	85	36	**0.003**	NS
Alternate	98	42(80.77%)	56(62.92%)	***P* = 0.036**	70(82.35%)	19(52.77%)	***P* < 0.001**	
Adjacent	42	10(19.23%)	32(35.95%)	***P* = 0.038**	14(16.47%)	16(44.44%)	**0.001**	
3:0/others	1	0(0.00%)	1(1.12%)	NS	1(1.18%)	1(2.78%)	NS	

*NS, not statistically significant. ^*a*^The comparison of segregation patterns in the same gender between the current study and Zhang et al. (2019a). The bold values of *P* value meant the statistical difference was observed.*

**TABLE 3:** Analysis of meiotic segregation patterns of trivalent structure in blastocysts according to carrier’s age.

Segregation	Total	Carrier’s age			Carrier’s age (Zhang et al., 2019a)			*P* value^*a*^
Patterns		<35 years *n* (%)	≥35 years *n* (%)	*P* value	<35 years *n* (%)	≥35 years *n* (%)	*P* value	
Overall	977	836	141	NS	483	121	NS	NS
Alternate	708	610(72.97%)	98(69.50%)	NS	322(66.67%)	89(73.55%)	NS	
Adjacent	263	221(26.44%)	42(29.79%)	NS	155(32.09%)	30(24.79%)	NS	
3:0/others	6	5(0.60%)	1(0.71%)	NS	6(1.24%)	2(1.65%)	NS	
**Female carriers**								
Overall	504	415	89	NS	288	36	NS	NS
Alternate	318	262(63.13%)	56(62.92%)	NS	160(55.55%)	19(52.77%)	NS	
Adjacent	182	150(36.14%)	32(35.95%)	NS	122(42.36%)	16(44.44%)	NS	
3:0/others	4	3(0.72%)	1(1.12%)	NS	6(2.08%)	1(2.77%)	NS	
**Male carriers**								
Overall	473	421	52	NS	195	85	NS	NS
Alternate	390	348(82.66%)	42(80.77%)	NS	162(83.07%)	70(82.35%)	NS	
Adjacent	81	71(16.86%)	10(19.23%)	NS	33(16.92%)	14(16.47%)	NS	
3:0/others	2	2(0.48%)	0(0.00%)	NS	0(0.0%)	1(1.18%)	NS	

*NS, not statistically significant. ^*a*^The comparison of segregation patterns in the same gender between the current study and Zhang et al. (2019a).*

In all enrolled carriers of our study, Rob(13;14) was the most frequent, followed by Rob(14;21), accounting for 55.3% (120/217) and 10.6% (23/217) respectively (Table 4). The data indicated that the rate of alternate segregation pattern in Rob(13;14) carriers was significantly higher (*P* = 0.010, OR = 1.74) than that in Rob(14;21) carriers, while the frequency of adjacent segregation pattern was lower (*P* = 0.032, OR = 0.63), especially in young carriers and male carriers. In the elder and female subgroups, there was no statistical difference.

**TABLE 4:** Analysis of meiotic segregation patterns in blastocysts between common Rob(13;14) and Rob(14;21).

Segregation	Translocation type	
Patterns	Rob(13;14)	Rob(14;21)
**Overall**	575	132
Alternate	443(77.04%)	87(65.90%)
Adjacent	130(22.61%)	42(31.82%)
3:0/others	2(0.35%)	3(2.27%)
**Age < 35 years**		
Overall	493	116
Alternate	384(77.89%)	76(65.52%)
Adjacent	108(21.91%)	37(31.90%)
3:0/others	1(0.20%)	3(2.59%)
**Age ≥ 35 years**		
Overall	82	16
Alternate	59(71.95%)	11(68.75%)
Adjacent	22(26.83%)	5(31.25%)
3:0/others	1(1.22%)	0(0.0%)
**Male carriers**		
Overall	314	87
Alternate	260(82.80%)	50(57.47%)
Adjacent	53(16.88%)	35(40.23%)
3:0/others	1(0.32%)	2(2.30%)
**Female carriers**		
Overall	261	45
Alternate	183(70.11%)	37(82.22%)
Adjacent	77(29.50%)	7(15.55%)
3:0/others	1(0.38%)	1(2.22%)

### Genome Aneuploidy Analysis

**FIGURE 2:**
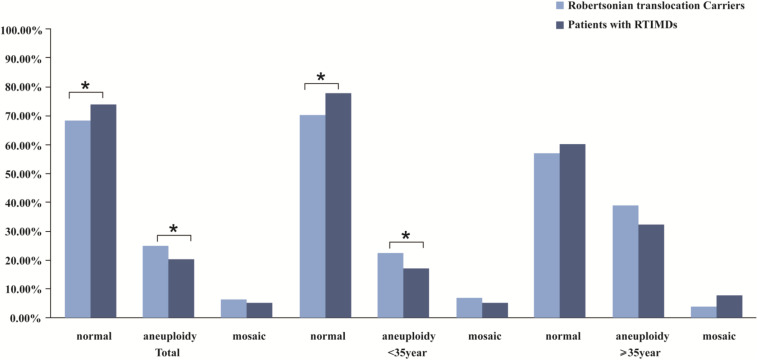
The comparison of meiotic segregation products of non-translocation chromosomes in blastocysts between Robertsonian translocation carriers and patients with RTMIDs. Legend: The trivalent structure could significantly increase the frequencies of chromosome aneuploidy 1.30 times in Robertsonian translocation carriers compared with patients with RTMIDs (*P* = 0.026), especially for the male and young carriers group (*P* = 0.030, OR = 1.35 and *P* = 0.012, and OR = 1.40). By contrast, the mosaic chromosome aneuploidy presented no statistical difference between the two groups. For elder carriers, no difference was observed between different subgroups.

**FIGURE 3:**
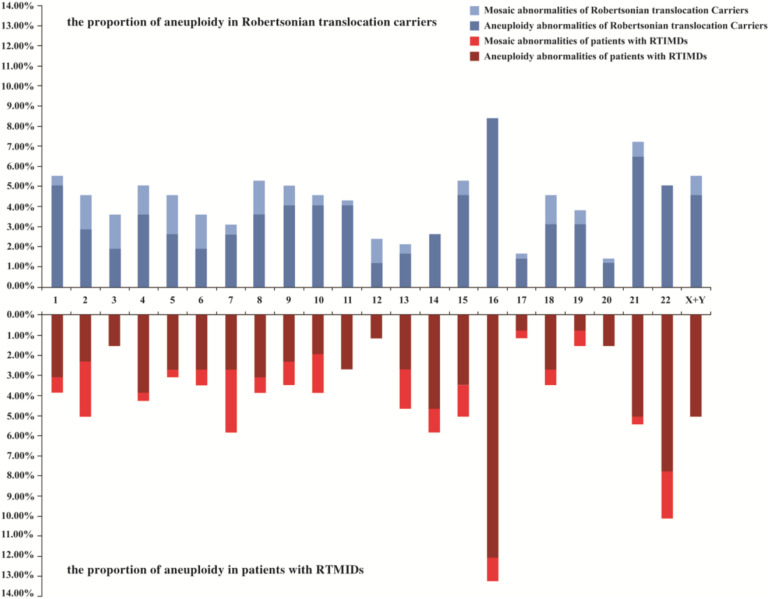
The distribution of non-translocation chromosome abnormalities in blastocysts of Robertsonian translocation carriers and all chromosome abnormalities in blastocysts of patients with RTMIDs. Legend: Horizontal axis represents chromosome 1-XY, vertical axis represents the proportion of aneuploidy and mosaic aneuploidy abnormalities. For Robertsonian translocation carriers, 977 blastocysts were included, and for patients with RTMIDs, 875 blastocysts were included in this study. RTMIDs, risk of transmitting monogenic inherited disorders.

We further evaluate the effect of trivalent structure on non-translocation chromosomes during meiosis. The segregation products of non-translocation chromosome in translocation carriers and those of patients with RTMIDs were compared. According to the results, the trivalent structure could significantly increase the frequencies of chromosome aneuploidy 1.30 times in Robertsonian translocation carriers compared with patients with RTMIDs (*P* = 0.026, OR = 1.30), especially for the male and young carriers group (*P* = 0.030, OR = 1.35 and *P* = 0.012, OR = 1.40). However, the mosaic chromosome aneuploidy presented no statistical difference between the two groups (Table 5 and Figure 2), the mosaic embryo was defined as having a chromosomal ratio level exceeding 20% of the aneuploid cells (Zhang et al., 2019b). For elder carriers, no difference was observed among different subgroups. The distribution of non-translocation chromosome abnormalities in Robertsonian translocation carriers and abnormalities across the genome in patients with RTMIDs was shown in Figure 3.

**TABLE 5:** The comparison of segregation products of non-translocation chromosomes in blastocysts between translocation carriers and patients with RTIMDs.

	Non-translocation	Robertsonian translocation	Patients with	*P*-value	OR
	Chromosome products	Carriers (%)	RTIMDs *n* (%)		
Overall		977	785		
	Normal	671(68.68%)	584(74.39%)	***P* = 0.009**	**0.76**
	Aneuploidy^*a*^	242(24.77%)	159(20.25%)	***P* = 0.026**	**1.30**
	Mosaic	64(6.55%)	42(5.35%)	NS	NS
Age < 35 year	Normal	590(70.66%)	485(78.10%)	***P* = 0.001**	**0.68**
	Aneuploidy	187(22.40%)	106(17.07%)	***P* = 0.012**	**1.40**
	Mosaic	58(6.95%)	30(4.83%)	NS	NS
Age ≥ 35 year	Normal	81(57.04%)	99(60.36%)	NS	NS
	Aneuploidy	55(38.73%)	53(32.31%)	NS	NS
	Mosaic	6(4.23%)	12(7.31%)	NS	NS
Male	Normal	319(67.44%)	584(74.39%)	***P* = 0.010**	**0.71**
	Aneuploidy	121(25.58%)	159(20.25%)	***P* = 0.030**	**1.35**
	Mosaic	33(6.98%)	42(5.35%)	NS	NS
Age < 35 year	Normal	288(68.41%)	485(78.10%)	***P* = 0.001**	**0.61**
	Aneuploidy	102(24.22%)	106(17.07%)	***P* = 0.006**	**1.55**
	Mosaic	31(7.36%)	30(4.83%)	NS	NS
Age ≥ 35 year	Normal	31(59.61%)	99(60.36%)	NS	NS
	Aneuploidy	19(35.54%)	53(32.31%)	NS	NS
	Mosaic	2(3.85%)	12(7.31%)	NS	NS
Female	Normal	352(69.84%)	584(74.39%)	NS	NS
	Aneuploidy	121(24.01%)	159(20.25%)	NS	NS
	Mosaic	31(6.15%)	42(5.35%)	NS	NS
Age < 35 year	Normal	302(72.95%)	485(78.10%)	NS	NS
	Aneuploidy	85(20.53%)	106(17.07%)	NS	NS
	Mosaic	27(6.52%)	30(4.83%)	NS	NS
Age ≥ 35 year	Normal	50(55.56%)	99(60.36%)	NS	NS
	Aneuploidy	36(40.00%)	53(32.31%)	NS	NS
	Mosaic	4(4.44%)	12(7.31%)	NS	NS

*NS, not statistically significant. ^*a*^Aneuploidy includes single aneuploidy/segmental aneuploidy/aneuploidy + segmental aneuploidy/aneuploidy + mosaic/segmental aneuploidy + mosaic/aneuploidy + segmental aneuploidy + mosaic. The bold values of *P* value meant the statistical difference was observed.*

## Discussion

With the development of genetic techniques, CCS with microarray or next-generation sequencing (NGS) can be performed and largely enhance the clinical outcomes of assisted reproduction for chromosomal rearrangements carriers. Meanwhile, even the embryos with translocation karyotype or normal karyotype can be distinguished before transfer in our previous publications (Zhang et al., 2017, 2019c). In the present study, SNP-array technology was utilized and the molecular karyotype of each embryo was analyzed to identify the chromosome aneuploidies related to translocation and *de novo* abnormalities. Afterward, meiotic segregation patterns were determined with molecular karyotype. Numerous products could be generated and alternate segregation products were the most frequent, followed by adjacent product and 3:0 product, which were consistent with previous studies (Mahjoub et al., 2011; Bernicot et al., 2012; Pylyp et al., 2013; Wang et al., 2017; Zhang et al., 2019a). However, the outcomes of different carriers also presented meiotic heterogeneity (Bint et al., 2011; Ko et al., 2013; Zhang et al., 2019a).

To determine the factors that affect meiotic segregation patterns, the segregation patterns were analyzed based on the carrier’s gender, age and translocation type in this study. When the influence of carrier’s gender was compared, we found that the male carriers had a much higher proportion of alternate segregation containing normal or balanced chromosome contents than the female carriers, and had a much lower frequency of adjacent segregation pattern, This is consistent with the findings of other published studies performed using FISH or array-CGH method (Bint et al., 2011; Ko et al., 2013; Zhang et al., 2019a). When conducting analysis of the meiotic segregation products of trivalent structure by carrier’s age, the distribution of segregation products displayed no difference between young and elder carriers, which was consistent with previous studies (Ko et al., 2013; Zhang et al., 2019a). Through conducting stratified analysis by gender, the distribution still exhibited no statistical difference. Consistent with previous research, the carrier’s age had no effect on meiotic segregation heterogeneity (Zhang et al., 2019a). Rob(13;14) and Rob(14; 21) were common translocation types, accounting for 55.3% (120/217) and 10.6% (23/217) respectively, in the current research. When conducting the comparison of translocation type to the impact on meiotic segregation patterns, the results demonstrated that the rate of alternate segregation pattern in Rob(13;14) carriers was significantly higher than that in Rob(14;21) carriers, especially in young and male carriers. Furthermore, sperm-FISH studies revealed that different translocation types displayed different proportion of alternate segregation, ranging from 76 to 89%. Besides, the sperm aneuploidy that was not involved in a particular translocation exhibited an increased frequency, which might increase the probability of chromosomal abnormalities in embryos (Hajlaoui et al., 2018; Wiland et al., 2020). On the contrary, Huang et al. (2010) found that genders of the carriers and translocation type have no effect on the meiotic segregation of the trivalent structure. The difference could be due to the limited sample number and characteristics of translocation carriers that were involved in the present study, thus resulting in insufficient analysis of the impact factors on segregation modes.

Our previous study revealed that meiotic segregation modes in reciprocal translocation carriers can be affected by the location of translocation breakpoints, carrier’s gender, carrier’s age and chromosome type (Zhang et al., 2018). In cases with reciprocal translocations involving an acrocentric chromosome, male carriers have a significantly higher proportion of adjacent-1 segregation product than female carriers, whereas the 3:1 and 4:0/other meiotic segregation patterns were lower than those in female carriers. Alternate and adjacent-2 segregation products showed no statistical difference between different genders. In addition, the study of Wang et al. (2019) indicated that the segregation modes in blastocysts were affected by the presence of acrocentric chromosomes and terminal breakpoints. These results suggested that meiotic heterogeneity between Robertsonian translocations and reciprocal translocation carriers was inconsistent and translocation type played an important role.

The phenomenon of ICE originates from meiotic disturbances in the proper pairing and disjunction of other chromosomes that are not involved in the translocation (Burgoyne et al., 2009). Consequently, ICE might significantly increase the numerical abnormalities of non-translocation chromosomes. To evaluate the effect of trivalent structure on genome stability or ICE during meiosis in the present study, 134 patients with RTMIDs were collected. The abnormalities of non-translocation chromosomes from translocation carriers were compared with those from patients with RTMIDs. We found that trivalent structures could increase the frequency of chromosome abnormalities 1.30 times in translocation carriers compared with patients with RTMIDs, especially for male (1.35 times) and young (1.40 times) carriers. When stratified analysis was performed by the abnormal type of aneuploidy or mosaic anomalies, the frequencies of aneuploidy were comparable to the global distribution. However, no significant difference was observed in mosaic anomalies. Additionally, there also existed no difference in both aneuploidy and mosaic anomalies in elder carrier subgroup. Possibly, advanced age revealed a more important role in forming abnormal chromosomes than trivalent structure. As reported, increasing maternal age contributed to the increased incidence of aneuploidies (Munne and Cohen, 2017; Pinheiro et al., 2019). The ICE phenomenon found in our research was consistent with previous studies, in which chromosomes were analyzed by FISH method (Anton et al., 2010; Mahjoub et al., 2011; Wang et al., 2017). ICE phenomenon would be instructive to explain the additional chromosome abnormalities and the large amounts of abnormal embryos. Most importantly, we also compared the distribution of chromosome abnormalities between translocation carriers and patient with RTMIDs, which is roughly consistent with the research made by Rodriguez-Purata et al. (2015), in which aneuploidy abnormalities of chromosome 16, 21, and 22 were the most common.

Moreover, our data would be useful in predicting the probability of diploid embryos and to counsel appropriately for translocation carriers undergoing PGT-SR. To our knowledge, this is the first report that simultaneously describes the factors impacting meiotic segregation and the effect of trivalent structure on genome aneuploidy. We also demonstrated that translocation type contributed to the meiotic heterogeneity of trivalent structure in blastocysts. In addition, this study explored the effect of trivalent structure on genome aneuploidy by analyzing segregation products of blastocysts from translocation carriers and patients with RTMIDs, which were used as comparable control samples. Apart from that, the larger sample size in our research is considered as another advantage. However, this study also has some limitations. First, this was a retrospective research and sample selection bias was inevitable. Second, the parental origin of abnormal chromosomes could not be identified. There is a chance that monosomies or trisomies of chromosomes involved in translocation at the blastocyst stage were *de novo* from the spouse who has normal karyotype or were introduced during early mitotic divisions after fertilization.

In conclusion, our results suggest that meiotic segregation heterogeneity of trivalent structure is linked with the carrier’s gender and translocation type, and is independent of the carrier’s age.

## Data Availability Statement

The original contributions presented in the study are included in the article/Supplementary Material, further inquiries can be directed to the corresponding author/s.

## Ethics Statement

Written informed consent was obtained from each family before the start of PGT cycle. The study protocol was approved by the Ethics Committee for Human Subject research of the Obstetrics and Gynecology Hospital, Fudan University.

## Author Contributions

SZ, XS, and CX designed the research and wrote the manuscript. SZ, CL, JW, SjZ, JZ, MX, YX, JF, YS, and XS executed the research. SZ, CL, MX, and YX performed the analysis. SZ, SjZ, JZ, and MX performed the microarray experiments. JZ performed cytogenetic experiments of amniotic fluid cell and blood. JF and YS performed the intra-cytoplasmic sperm injection and blastocyst biopsy experiments. JW, XS, and CL collected the cases. XS and CL directed the critical discussion of the manuscript. All authors approved the final manuscript.

## Conflict of Interest

The authors declare that the research was conducted in the absence of any commercial or financial relationships that could be construed as a potential conflict of interest.
