# Supplementary material for: Meiotic Heterogeneity of Trivalent Structure and Interchromosomal Effect in Blastocysts With Robertsonian Translocations
Source: Front Genet. 2021 Feb 16;12:609563. doi: 10.3389/fgene.2021.609563 (PMC7928295; doi:10.3389/fgene.2021.609563)
Supplement: Supplementary file 1 [file Table_1.docx]

| **Supplemental Table 1. Semen parameters of men with Robertsonian translocation and men with normal karyotype** | | | |
| --- | --- | --- | --- |
| **Parameters** | **Men with normal karyotype** | **Men with Robertsonian translocation** | **P-value** |
| Sperm concentration(×10^6^/ml) | 46.71±27.22 | 37.86±27.54 | **0.022** |
| Sperm progressive motility (%) | 41.32±19.55 | 36.69±20.31 | **0.015** |
| Semen volume(ml) | 2.85±1.22 | 2.77±1.03 | NS |
| Total sperm(10^6^/ejaculate) | 131.42±92.90 | 118.32±94.43 | NS |
| Total progressively motile sperm | 56.11±54.58 | 52.34±52.87 | NS |
| (10^6^/ejaculate) |  |  |  |
| NS= not statistically significant | | | |
