# Supplementary material for: Meiotic Heterogeneity of Trivalent Structure and Interchromosomal Effect in Blastocysts With Robertsonian Translocations
Source: Front Genet. 2021 Feb 16;12:609563. doi: 10.3389/fgene.2021.609563 (PMC7928295; doi:10.3389/fgene.2021.609563)
Supplement: Supplementary file 2 [file Table_2.DOCX]

**Supplemental Table 2. Analysis of segregation patterns of Rob(13;14) according to carrier’s gender**

| **Segregation** | **Total** | **Carrier’s gender** | | **P-value** |
| --- | --- | --- | --- | --- |
| **patterns** |  | **Male n (%)** | **Female n (%)** |  |
| **Overall** | 575 | 314 | 261 | **P<0.001** |
| Alternate | 443 | 260(82.80%) | 183(70.11%) | **P<0.001** |
| Adjacent | 130 | 53(16.88%) | 77(29.50%) | **P<0.001** |
| 3:0/others | 2 | 1(0.32%) | 1(0.38%) | NS |
| **Age <35years** |  |  |  |  |
| Overall | 493 | 277 | 216 | **P<0.001** |
| Alternate | 384 | 232(83.03%) | 152(70.37%) | **P<0.001** |
| Adjacent | 108 | 44(15.88%) | 64(29.63%) | **P<0.001** |
| 3:0/others | 1 | 1(0.36%) | 0(0.0%) | NS |
| **Age ≥35years** |  |  |  |  |
| Overall | 82 | 37 | 45 | NS |
| Alternate | 59 | 28(75.68%) | 31(68.89%) | NS |
| Adjacent | 22 | 9(24.32%) | 13(28.89%) | NS |
| 3:0/others | 1 | 0(0.00%) | 1(2.22%) | NS |
