# Supplementary material for: Meiotic Heterogeneity of Trivalent Structure and Interchromosomal Effect in Blastocysts With Robertsonian Translocations
Source: Front Genet. 2021 Feb 16;12:609563. doi: 10.3389/fgene.2021.609563 (PMC7928295; doi:10.3389/fgene.2021.609563)
Supplement: Supplementary file 3 [file Table_3.DOCX]

**Supplemental Table 3. Analysis of segregation patterns of Rob(13;14) according to carrier’s age**

| **Segregation** | **Total** | **Carrier’s age** | | | **P-value** |
| --- | --- | --- | --- | --- | --- |
| **patterns** |  | **<35 years n (%)** | | **≥35 years n (%)** |  |
| **Overall** | 575 | 493 | 82 | |  |
| Alternate | 443 | 384(77.89%) | 59(71.95%) | | NS |
| Adjacent | 130 | 108(21.91%) | 22(26.83%) | | NS |
| 3:0/others | 2 | 1(0.20%) | 1(1.22%) | | NS |
| **Female carrier** |  |  |  | |  |
| Overall | 261 | 216 | 45 | | NS |
| Alternate | 183 | 152(70.37%) | 31(68.89%) | | NS |
| Adjacent | 77 | 64(29.63%) | 13(28.89%) | | NS |
| 3:0/others | 1 | 0(0.0%) | 1(2.22%) | | NS |
| **Male carrier** |  |  |  | |  |
| Overall | 314 | 277 | 37 | | NS |
| Alternate | 260 | 232(83.03%) | 28(75.68%) | | NS |
| Adjacent | 53 | 44(15.88%) | 9(24.32%) | | NS |
| 3:0/others | 1 | 1(0.36%) | 0(0.00%) | | NS |
